# Clinical outcomes for patients with synovial sarcoma of the hand

**DOI:** 10.1186/2193-1801-3-649

**Published:** 2014-11-03

**Authors:** Hidetatsu Outani, Kenichiro Hamada, Kazuya Oshima, Susumu Joyama, Norifumi Naka, Nobuhito Araki, Takafumi Ueda, Hideki Yoshikawa

**Affiliations:** Department of Orthopaedic Surgery, Osaka University Graduate School of Medicine, 2-2, Yamada-oka, Suita, Osaka 565-0871 Japan; Musculoskeletal Oncology Service, Osaka Medical Center for Cancer and Cardiovascular Diseases, 1-3-3 Nakamichi, Higashiknari, Osaka 537-8511 Japan; Department of Orthopaedic Surgery, Osaka National Hospital, 2-1-14 Hoenzaka, Chuo-ku, Osaka 540-0006 Japan

**Keywords:** Synovial sarcoma, Soft tissue sarcoma, Hand, Hand preservation

## Abstract

**Purpose:**

Soft tissue sarcoma of the hand is rare, and one of the most common histological diagnosis is synovial sarcoma. We report the clinical outcomes of patients with synovial sarcoma of the hand and discuss treatment strategies.

**Methods:**

We reviewed five patients with synovial sarcoma of the hand treated at our institutions from 1983 to 2013. The mean patient age at the time of diagnosis was 36.6 years (range, 20–62 years). Two patients underwent marginal excision after neoadjuvant chemotherapy, followed by radiation therapy, one underwent wide local excision and two received chemotherapy and radiation therapy.

**Results:**

The average duration of follow-up for all patients was 88.2 months (range, 14–218 months). Two patients continuously remained disease free, two experienced local recurrence requiring additional surgery and then showed no evidence of disease, and one who had distant metastasis at diagnosis died of the disease. No patients developed lymph node metastasis. The estimated 5-year overall survival was 80%.

**Conclusions:**

Our case series suggests that patients with localised synovial sarcoma of the hand may have favourable outcomes. Wide excision or marginal excision, followed by radiation therapy combined with chemotherapy, represent acceptable treatment strategies for synovial sarcoma of the hand. Regional lymph node dissection does not seem to be essential for synovial sarcoma of the hand.

## Introduction

Soft tissue sarcoma of the hand is rare, with an incidence of approximately 2%–4% of limb and trunk tumours (Pradhan et al. 2008; McPhee et al. 1999). The histological types of soft tissue sarcoma in the hand are limited (Pradhan et al. 2008). The most frequent diagnoses are synovial sarcoma, clear cell sarcoma and epithelioid sarcoma (Pradhan et al. 2008; Brien et al. 1995; Puhaindran et al. 2011). Most sarcomas of the hand are currently treated with a multimodality strategy including limb-sparing surgery (Hsu et al. 2007). For epithelioid sarcoma, an exceptionally wide excision and regional lymph node dissection are recommended because of the increased risk of metastasis to the lymph nodes (Brien et al. 1995; Kawai et al. 2002). Pradhan et al. reported that clear cell sarcoma subtype is correlated with a poor survival rate due to the high risk of local recurrence (Pradhan et al. 2008). As mentioned above, the treatment strategy for soft tissue sarcoma of the hand may differ among tumour subtypes because of differences in local recurrence and metastatic potential. The purpose of this study was to review the clinical outcomes of patients with synovial sarcoma of the hand treated at our institutions, and to discuss appropriate treatment strategies for synovial sarcoma of the hand.

## Materials and methods

We performed a retrospective review of all patients with a synovial sarcoma of the hand who were treated at our institutions over a period of 30 years from 1983 to 2013. Among 137 synovial sarcoma patients, five with a synovial sarcoma of the hand were identified from the institutional database (4%). The clinical characteristics of the patients are summarised in Table 1. All patients were male with the mean age of 36.6 years (range, 20–62 years), and all patients had tumours that were over the dorsum of the hand. All patients gave informed consent for their data to be included in this study. The ethical committee of our institute gave institutional review board approval to this retrospective study.

**Table 1:** **Patient summary**

Case/sex/age	Presentation	Location	Maximum dimension(cm)	AJCC stage	CTx	RTx	Follow-up(months)
1/M/45	After previous surgery	Dorsum	4	IIA	Yes	Yes	63
2/M/23	After previous surgery	Dorsum	3	IIA	No	No	218
3/M/62	Primary	Dorsum	8	IIB	Yes	Yes	43
4/M/20	Primary	Dorsum	6	IV	Yes	Yes	14
5/M/33	After previous surgery	Dorsum	3	IIA	Yes	Yes	103
**Case**	**Procedure**	**Margins**	**Local recurrence**		**Distant metastases**		**Status**
1	Marginal excision	Negative	Yes		No		NED
2	Wide excision with free tendon graft	Negative	No		No		CDF
3	Marginal excision with IORTG	Negative	No		No		CDF
4	No operation	-	(Persistent disease)		Yes		DOD
5	*Double ray amputation	Negative	Yes		Yes		NED

CTx, chemotherapy; RTx, radiation therapy.

NED, no evidence of disease; CDF, continuous disease free; DOD, dead of disease; IORTG, intraoperative extracorporeal autogenous irradiated tendon grafts; *operation was performed after local recurrence.

All patients were staged with computed tomography (CT) scanning of the chest and magnetic resonance imaging (MRI) of the hand. The American Joint Committee on Cancer (AJCC) staging system was used to stage the tumours (Greene et al. 2002). Three patients had AJCC stage IIA tumours, one had a stage IIB tumour and one had a stage IV tumour.

**Figure 1:**
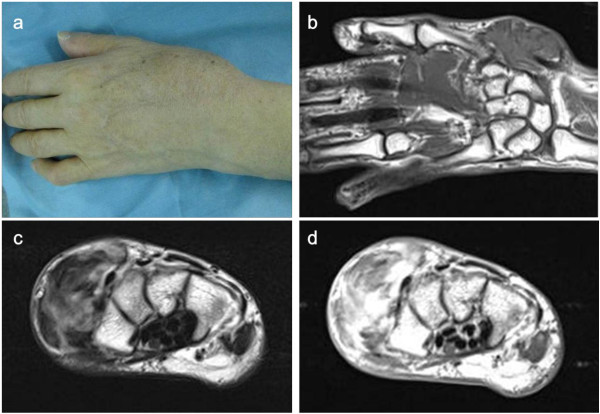
**Representative case: case 3. (a)** A 62-year-old male patient with synovial sarcoma in the dorsoradial aspect of the hand. **(b)** Magnetic resonance imaging (MRI) of the tumour showed isointensity on a T1 image, **(c)** inhomogeneous high on a T2 image and **(d)** enhancement with the use of gadolinium. The mass spanned the first, second and third compartments of the extensor tendon sheath but with no evidence of bone involvement.

Two patients underwent biopsy at our institutions (Cases 3 and 4). Of these two patients, one was treated with marginal excision after neoadjuvant chemotherapy, followed by radiation therapy (Figure 1), and the other patient who had distant metastasis at diagnosis refused surgery and received chemotherapy and radiation therapy. Three patients underwent inappropriate surgical excisions without wide safety margins in another hospital and required further treatment (Cases 1, 2 and 5). Of these three patients, one developed multiple local recurrences and underwent marginal excision to avoid hand amputation and preserve hand function after neoadjuvant chemotherapy followed by radiation therapy, one underwent wide excision with free tendon graft reconstruction, and the third refused surgery and received chemotherapy and radiation therapy. From 1983 to 1996, the chemotherapy regimen was dacarbazine, vincristine, nimustine, and interferon β (dacarbazine, 140 mg/m^2^/day over 1–5 days; vincristine, 1.5 mg/m^2^ only on the first day; nimustine, 80 mg/m^2^ only on the first day and interferon β, 6 × 10^6^ U/day for 1–5 days). After 1997, the regimen was a combination of doxorubicin and ifosfamide (doxorubicin, 30 mg/m^2^/day for 1–2 days and ifosfamide, 2.5 g/m^2^/day for 1–4 days) or high-dose ifosfamide (3 g/m^2^/day for 1–5 days).

## Results

The average duration of follow-up for all patients was 88.2 months (range, 14–218 months). The presented symptoms included pain and swelling in one patient and swelling only in four patients. The mean duration of symptoms before diagnosis was 8.2 months (range, 1-12 months).

**Figure 2:**
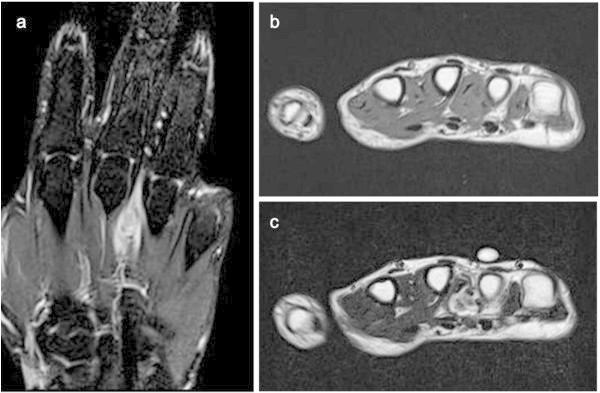
**Recurrent synovial sarcoma in the dorsum of the hand: case 5. (a)** MRI of the tumour showed high intensity on T1 fat sat image, **(b)** isointensity on a T1 image and **(c)** high intensity on a T2 image. The mass spanned the third and fourth metacarpal bones.

Two patients developed local recurrence, and both were treated with unplanned excisions at another hospital. One (Case 1) received repeated marginal excision to avoid hand amputation, followed by radiation therapy, and the other (Case 5) underwent double ray amputation after local recurrence, because the patient already had received radiation therapy and could not undergo marginal excision followed by radiation therapy (Figure 2). In addition, the latter patient developed metastasis to the lung after initial presentation and underwent pulmonary metastasectomy. No patients developed lymph node metastasis.

During the previous follow-up, two patients continuously remained disease free, two showed no evidence of disease after treatment for local recurrence and one died of distant metastases. The estimated 5-year overall survival was 80%.

## Discussion

Synovial sarcoma accounts for 5%–10% of all soft tissue sarcomas, frequently affecting adolescents and young adults and predominantly occurs in the extremities (Goldblum et al. 2013). Synovial sarcoma arising in the upper extremities, which accounts for approximately 10%–15% of all cases, are fairly evenly distributed among the forearm–wrist region, shoulder, elbow–upper arm region and hand (Goldblum et al. 2013). In our study, 4% of synovial sarcomas were observed in the hand, and this incidence is similar to that of all extremity-related sarcomas (Pradhan et al. 2008; McPhee et al. 1999).

The typical presentation of synovial sarcoma of the hand is a slow-growing mass. Because of the rarity of the soft tissue sarcoma of the hand, 38%–95% of these tumours are treated by unplanned excision before referral to a specialist oncological center (Pradhan et al. 2008; Lin et al. 2002). Pradhan et al. reported that no significant correlation exists between previous surgical excision and the risk of local recurrence or overall survival (Pradhan et al. 2008). However, Talbot et al. reported that an unplanned excision represents a larger operation requiring flap coverage in affected patients (Talbot et al. 2008). In the present report, three of the five patients had been treated with an unplanned excision. One of the three patients underwent free tendon graft reconstruction, but no patients required flap coverage. Moreover, all of these three patients experienced a favourable outcome after multimodal treatment. In cases of large skin defects after surgical excision, skin grafting and flap coverage are important options. The radial forearm flap and the reversed radial forearm flap can be used to effectively cover large soft tissue defects in the hand (Talbot et al. 2008; Ferguson 2005). Tendons can often be resected with the tumours and reconstructed with free tendon grafts from the palmaris longus. However, when multiple tendons were surrounded by the tumours, we previously re-implanted intraoperative extracorporeal autogenous irradiated tendon grafts to restore hand function (Araki et al. 1999).

Because of its complex anatomy and limited tissue volume, soft tissue sarcoma of the hand has a tendency to rapidly spread between compartments which makes excision with a wide margin difficult (Pradhan et al. 2008; Kawai et al. 2002). Unfortunately, the minimal acceptable margin has not been established (Siegel et al. 2007). Talbert et al. reported the lack of a survival benefit from amputation in patients with soft tissue sarcoma of the distal extremities and recommended conservation surgery and radiation therapy (Talbert et al. 1990). In contrast, Brien et al. reported that adjuvant radiotherapy could not compensate for positive surgical margins (Brien et al. 1995). Two independent investigators emphasised the importance of negative surgical margins for local control and prevention of metastatic disease (Pradhan et al. 2008; Puhaindran et al. 2011). However, those studies included various tumour sub-types. Few reports have focused on synovial sarcoma of the hand (Dreyfuss et al. 1986; Michal et al. 2006), and the best treatment strategy for synovial sarcoma of the hand remains unknown. Synovial sarcoma is relatively chemosensitive (Canter et al. 2008; Rosen et al. 1994). Therefore, our approach has been to perform marginal excision, avoiding amputation of the entire hand, followed by radiation therapy combined with chemotherapy to preserve hand function and to reduce the risk of local recurrence or distant failure when it is difficult to achieve wide local excision. However, we assume that amputation is still required when an important neurovascular structure cannot be preserved or when local recurrence has occurred after postoperative radiotherapy. Some authors have reported the importance of regional lymph node dissection for some sub-types of sarcoma of the hand (Brien et al. 1995; Kawai et al. 2002). Although the small number of patients is a limitation of the present study, no patients developed lymph node metastasis in this study. In a previous case series, Brien et al. described the clinical features of soft tissue sarcoma of the hand (Brien et al. 1995), and of the 23 patients in that series, 8 had synovial sarcomas and none of them developed lymph node metastasis. Therefore, we believe that regional lymph node dissection is not essential for patients with synovial sarcoma of the hand.

The favourability of prognosis in patients with synovial sarcoma of the hand is controversial. Canter et al. reviewed 255 patients with localised synovial sarcoma of all anatomic sites and reported a 5-year disease-specific survival of 72%; an upper extremity location was associated with favourable prognosis (Canter et al. 2008). Dreyfuss et al. reviewed 44 cases of synovial sarcoma of the hand that were treated over the period from 1934 to 1984 and reported a 5-year overall survival of only 18% (Dreyfuss et al. 1986). However, of those 44 cases, 13 patients (30%) were treated with radiation therapy and only two patients (5%) were treated with chemotherapy. In our previous 30-year series, the estimated 5-year overall survival was 80%. Improvements in pre-operative imaging and adjuvant therapies may have contributed to patient survival in our current series.

In conclusion, we report five cases of synovial sarcoma of the hand. The clinical outcomes of synovial sarcoma of the hand were favourable when distant metastasis was not present at diagnosis. Patient survival was similar to that for synovial sarcoma in all locations. Wide excision or marginal excision, followed by radiation therapy combined with chemotherapy, are acceptable treatment strategies for synovial sarcoma of the hand. Regional lymph node dissection does not seem to be essential for synovial sarcoma of the hand.
